# Dentofacial characteristics of oral breathers in different ages: a retrospective case–control study

**DOI:** 10.1186/s40510-015-0092-y

**Published:** 2015-07-15

**Authors:** Rosa Carrieri Rossi, Nelson José Rossi, Nelson José Carrieri Rossi, Hélio Kiitiro Yamashita, Shirley Shizue Nagata Pignatari

**Affiliations:** Division of Pediatric Otolaryngology, Federal University of Sao Paulo- UNIFESP Brasil, Rua Botucatu 740, 4 andar, V. Clementino, São Paulo, CEP:04023-062 Brazil; Educational Association of Brazil- FUNORTE/SOEBRAS, Rua Tijuco Preto 1694, Sao Paulo, Tatuapé CEP:03316-000 Brazil; Department of Diagnostic Imaging, Division of Otolaryngology and Head and Neck Imaging, Federal University of Sao Paulo- UNIFESP, Rua Botucatu 740, 4 andar, V. Clementino, São Paulo, CEP:04023-062 Brazil; Department of Otolaryngology and Head and Neck Surgery, Division of Pediatric Otolaryngology, Federal University of Sao Paulo- UNIFESP, Rua Botucatu 740, 4 andar, V. Clementino, São Paulo, CEP:04023-062 Brazil

**Keywords:** Mouth breathing, Oral breathing, Respiration

## Abstract

**Background:**

This study aimed to investigate the dental and skeletal variables associated with disturbances of craniofacial development in oral-breathing (OB) individuals and the probability that these variables are related to this condition.

**Methods:**

This is an observational retrospective case–control study of 1596 patients divided into three groups of age n1 5–12, n2 13–18, and n3 19–57 years. Radiographic, clinical, and models data were analyzed. The control group was consisted of nasal breathing (NB) individuals. Statistical analyses of the qualitative data were performed with *x*^2^ test to identify associations, and odds ratio (OR) tests were performed for the variables that the chi-square test (*x*^2^) identified an association.

**Results:**

In the descriptive analysis of the data, we observed that the class II malocclusion was the most frequent in the total sample, but when divided by age group and mode of breathing, there is a random division of these variables. In n1 group, class II, (OR = 2.02) short and retruded mandible (SM and RM) (OR = 1.65 and1.89) were associated with OB and it was considered a risk factor. In n2 group, class II (OR = 1.73), SM (OR = 1.87) and increased lower anterior height (ILAFH) (OR = 1.84) seemed to be associated and to be risk factors for OB. In the n1 group, decreased lower anterior facial height (DLAFH) and brachycephalic facial pattern (BP) seemed to be associated with NB and a protective factor against oral breathing.

**Conclusions:**

This study showed that dental and skeletal factors are associated with OB in children, and it seems that it becomes more severe until adolescence. But adults showed no associations between OB and skeletal factors, only in dental variables, indicating that there is no cause–effect relationship between the dental and skeletal factors and OB. The treatment of nose breathing patient should be multidisciplinary, since OB remains even when dental and skeletal factors slow down.

## Background

Although the importance of nasal breathing (NB) for craniofacial growth and harmonious development is well established, a large number of studies also address oral breathing (OB) and its role in craniofacial changes [1]. The difficulty in understanding the causes and effects of respiratory patterns on craniofacial growth is a reflex of the fact that several factors acting simultaneously can influence it, and there is often inaccuracy in the diagnosis of oral and nasal breathing [2–25].

Some authors have claimed that OB alters growth and facial development [1, 2, 4, 7, 10–13, 16, 19, 21, 22, 24, 25], but others disagree and believe that the altered growth of the dentofacial complex results from other factors that may predispose individuals to OB [2–7].

The literature has not yet clearly demonstrated the relationship between OB and the growth and development. Addressing these questions is important since patients with such respiratory disturbances more frequently exhibit chronic sleep apnea and higher mortality rates and are more likely to develop cardiovascular diseases [7].

This study aimed to investigate the dental and skeletal variables associated with disturbances of craniofacial development in oral breathing individuals and the probability that these variables are related to this condition.

## Methods

This observational retrospective case–control study was approved by the Ethics Committee of the Federal University of São Paulo, protocol number 103.275. Informed consent was obtained from all patients or legal guardians.

The study population (*N* = 1596) was based on a consecutive sample of oral and nose breathing patients of both sexes between ages 5 and 57 years who sought orthodontic treatment in an orthodontics clinic in the city of São Paulo between the years 2006 and 2012.

The sample was consecutive and consisted of a large number of subjects in an attempt to control errors through descriptive and inferential statistical tests. The survey was conducted by the same examiner in a uniform manner to ensure consistency and quality of results [1, 2, 8–11].

Initially, the 1596 patients (*N*) were divided in three groups according to age range (n1, n2, n3), as previous studies:

- n1- 5 to 12 years old (*n* = 523) child ^1,6,8,10,12,20^
- n2- 13 to 18 years old (*n* = 443) adolescent ^2,4,5,7,21^
- n3- 19 to 57 years old (*n* = 340) adult ^3,22,23^

All patients had previous examinations of the same diagnostic center consisting of plaster model and side and panoramic radiographs with McNamara’s cephalometric analysis [24]. Measures such as the size of the mandible (NM, RM, PM), the lower anterior facial height (NLAFH, ILAFH, DLAFH), the position of the maxilla (NMx, RMx, PMx), and mandible (NM, LM, SM), and facial pattern (NP, BP, DP) were evaluated [24, 25].

Dental characteristics were obtained by prior clinical examination and model analysis. The position of the first permanent molars followed the Angle classification [26]. Intermolar (IM) and intercanine (IC) widths (W), superior (S) and inferior (I) widths were obtained in orthodontic model with a digital caliper measuring the distance between the central fosse of molars and between the cusp tip of the canines [5, 6, 19].

The patients were divided by type of breathing presented, using the protocol described in previous studies [11]. According to their predominant breathing pattern history and the findings on clinical examination, patients were classified as either OB or NB. The OB was considered as a study group (SG) and the NB as a control group (CG).

The orthodontist performed the history and clinical examination including evaluation of lips protrusion or retrusion, and the otolaryngologist was responsible for the speech and audiologic evaluations. Patients and their guardians answered a questionnaire concerning the breathing habits, while awake, with respect to any permanency of breathing with the mouth open, oral breathing, nasal obstruction, oral malodor, hyponasal speech, and while asleep, considering snoring, sleep apnea [7], restless sleep, or hyper salivation. Most of the questions were yes or no items as the protocol [11].

Patients who reported harmful habits such as finger sucking, the use of a pacifier during sleep or during the day, patients who had been submitted to orthodontic treatment, and those whose complete record could not be verified, were excluded. The data were collected in an Excel (Microsoft, 2010) database.

Comparison of OB and NB with dental and skeletal variables was accomplished among the three age groups (n1, n2, n3).

Qualitative variables were described by means of tables showing the percentage of occurrences for each category. After the division of the groups according to exclusion criteria, each variable had different n, in the tables. Correlation analysis was made between the pattern of breathing and the following variables:

1. breathing mode and gender
2. breathing mode and Angle class
3. breathing mode and maxilla position
4. breathing mode and mandible position
5. breathing mode and mandible size
6. breathing mode and anterior-lower facial height
7. breathing mode and facial pattern

Variables OB and NB groups, separated by age (n1, n2, n3), were evaluated by the homogeneity test (chi-square-*x*^2^). Pearson correlations were selected for a reliability of 95 % and a significance level of *p* < 0.05. Odds ratio (OR) tests were performed for the variables that the chi-square test (*x*^2^) identified an association.

The purpose of these tests was to identify the variable associated with OB and to determine the relative influence of these variables on the risk (OR) of developing the disease*.* When evidence of a correlation was found, we concluded that the variable was dependent of the breathing pattern.

Quantitative variables were described using measures of central tendency (mean and median) and measures of dispersion as standard deviation (SD). The Student’s *t* test was used to investigate the possible associations between the quantitative variables with confidence interval (CI) 95 % in each of the three groups (n1, n2, n3). Means and SD for the OB and NB, and the upper and lower intermolar and intercanine widths (IMSW, IMIW, ICSW, ICIW), were checked with the Minitab program, version 14.

For rejecting the null hypothesis we fixed the significance level of 5 %.

## Results

**Fig. 1:**
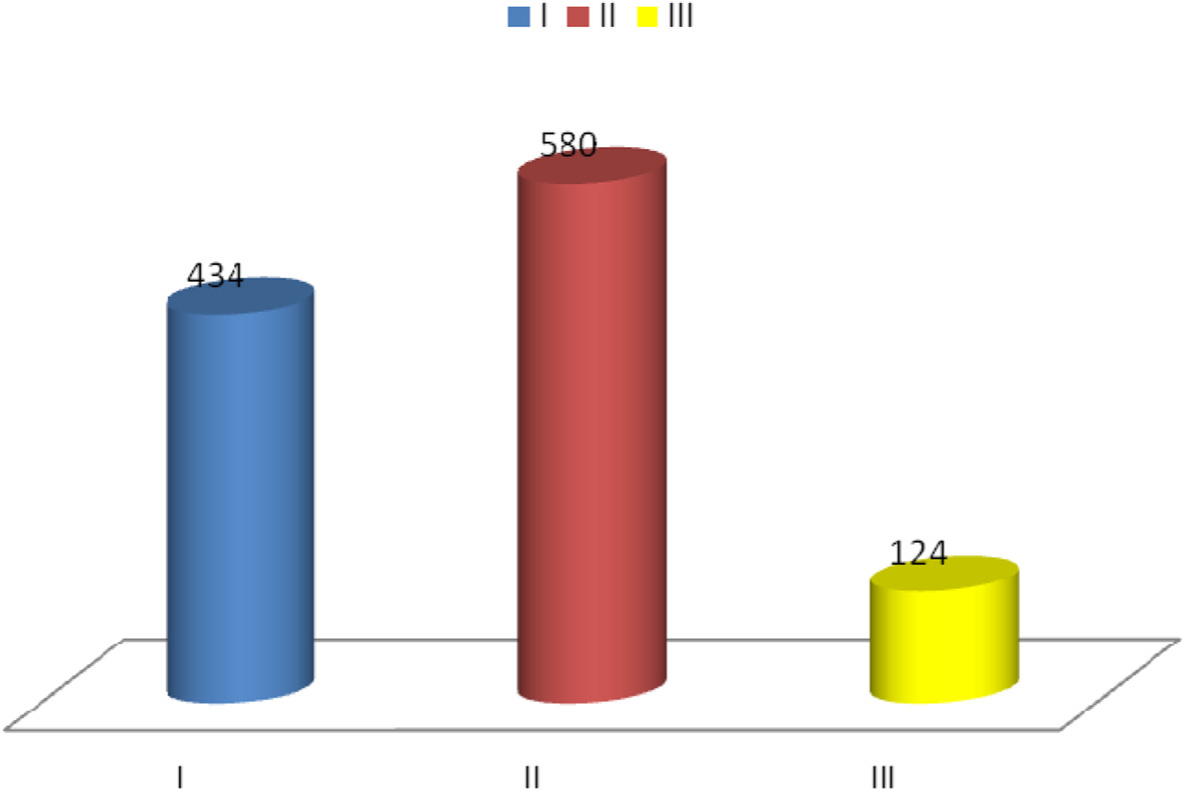
Angle malocclusion in the population study. There was a greater frequency of Angle Class II in the population study

**Fig. 2:**
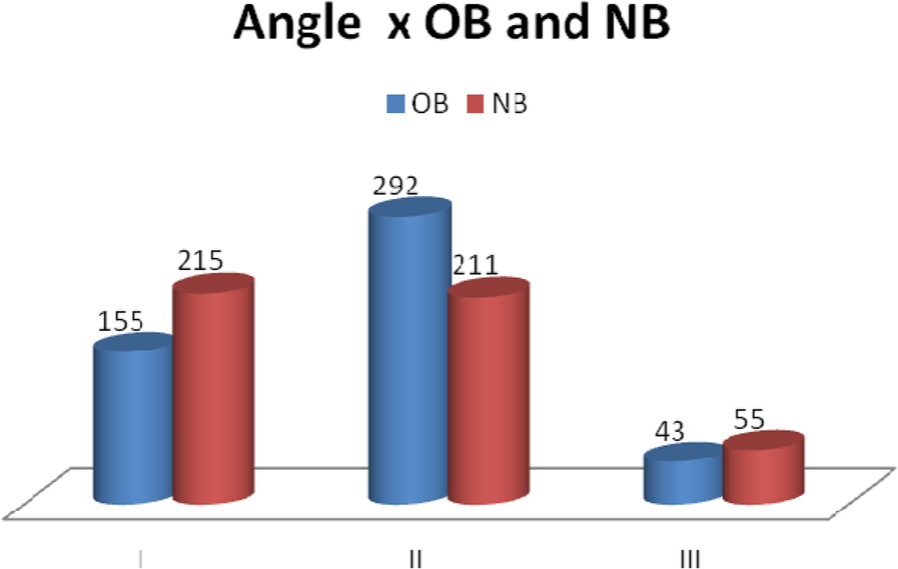
Frequency of Angle classes among OB and NB. There was an homogeneous distribution of Angle malocclusion classes between OB and NB

In the descriptive analysis of the data, we observed that the class II malocclusion was the most frequent in the total sample, but when divided by age group and mode of breathing, there is a random division of these variables (Figs. 1 and 2).

### Group 1 (n1 = 523) 5–12 years—Qualitative variables

For ages 5–12 years old, there was no evidence of association between gender, class III malocclusion, protruded (PMx) and retruded maxilla ( RMx), protruded mandible (PM), large mandible (LM), dolichocephalic pattern (DP), increase in lower anterior face height (ILAFH), and the type of breathing.

There was a significant association between class II malocclusion, retruded mandible (RM) and short mandible (SM); there was a significantly higher percentage of oral breathing with these variables. A higher percentage of NB with normal mandible (NM) was also observed.

An association between decreased LAFH (DLAFH) and the type of breathing was observed, as was a higher percentage of OB with normal LAFH (NLAFH) and a higher percentage of NB with DLAFH. Finally, in the association between the brachycephalic facial pattern (BP) and the type of breathing, a higher percentage of OB with normocephalic pattern (NP) and a higher percentage of NB with BP were found.

We observed odds ratio (OR) between class II malocclusion (OR = 2.02; CI (95 %) = (1.32, 3.09)), RM (OR = 1.89; CI (95 %) = (0.99, 3.60)), SM (OR = 1.65; CI (95 %) = (1.06, 2.58)) with OB.

We observed DLAFH and BP with NB, and these last two presented themselves as protective factors for the development of OB (OR = 0.44; CI (95 %) = (0.26, 0.77)) and (OR = 0,43, CI (95 %) = (0.24, 0.78)), respectively.

**Fig. 3:**
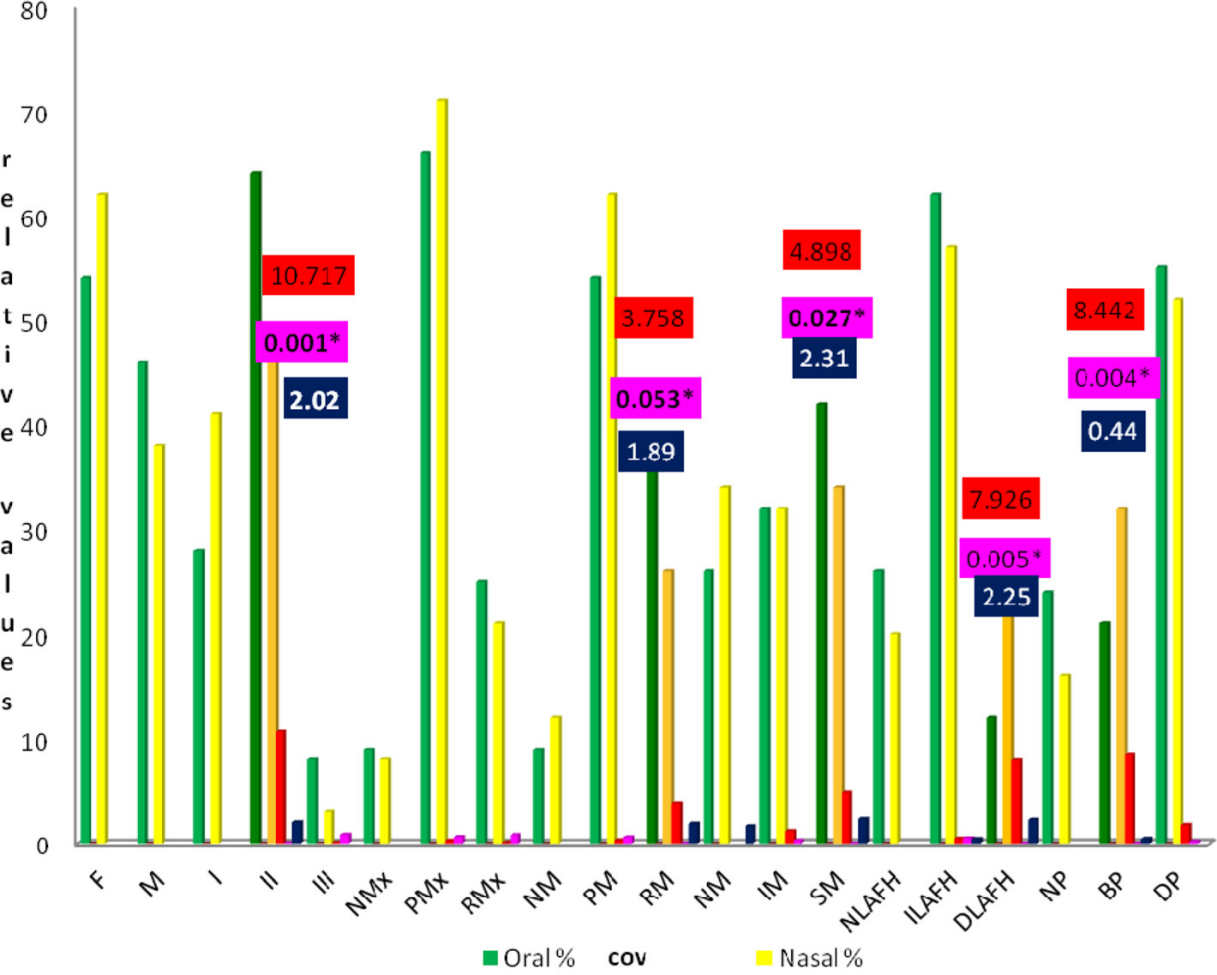
Intra-groups association values (Pearson’s *x* ^2^, OR) for qualitative variables, group n1 (523), 5 to 12 years of age, with OB and NB. *Significant values. The variables that showed significant associations and risk of disease were Class II, RM, SM. DLAFH and BP (protective factors)

The results of *x*^2^ and Pearson associations, OR for qualitative variables tests, in group 1 are shown in Table 1 and Fig. 3.

**Table 1:** Intra-group association tests (Pearson’s x^2^, odds ratio) of the qualitative variables—group n 1 (5 to 12 years of age)

Variables	OB (*n*)	OB %	NB (*n*)	NB %	Total	Pearson *x* ^2^	*p* value	DF	Lilihood *x* ^2^	OR	CI 95 %
F	152	54	130	63		2.423	1.11	1	2.43		
M	125	46	80	38							
*Total*	*277*		*210*		*487*						
I	67	28	73	41							
II	156	64	84	48		10.717	0.001*	1	10672	*2.02*	*(1.32:3.09)*
III	20	8	20	11		0.057	0.811	1	0.057		
*Total*	*243*		*177*		*420*						
NMx	25	9	17	8							
PMx	181	66	146	71		0.263	0.608	1	0.264		
RMx	68	25	42	21		0.067	0.795	1	0.067		
*Total*	*274*		*205*		*479*						
NM	25	9	25	12							
PM	149	54	126	62		0.297	0.586	1	0.297		
RM	100	37	53	26		3.758	0.053*	1	3,693		
*Total*	*274*		*204*		*478*						
NM	71	26	70	34							
IM	85	32	65	32		1.164	0.281	1	1.165	*1.65*	*(1.06; 2.58)*
SM	114	42	68	34		4.898	0.027*	1	4.897		
*Total*	*270*		*203*		*473*						
NLAFH	70	26	41	20							
ILAFH	168	62	115	57		0.456	0.499	1	0.459	*2.31*	*(1.28; 4.15)*
DLAFH	34	12	46	23		7.926	0.005*	1	7.957	*0.43*	*(0.24; 0.78)*
*Total*	*272*		*202*		*474*						
NP	65	24	33	16							
BP	56	21	64	32		8.442	0.004*	1	1.746	*0.44*	*(1.30; 3.90)*
DP	146	55	106	52		1.746	0.186	1	1.767	*3.25*	*(0.26; 0.77)*
*Total*	*267*		*203*		470						

*Significant values

The values with (*) and in italics represent significant values

### Quantitative variables

There was no evidence of a difference between the mean ages of the OB and NB in group 1. No differences between the means of the upper and lower intermolars widths were detected between the OB and NB. No evidence of differences in the means of the upper and lower intercanine widths were found.

Summaries of the values of the middle range are shown in Table 2.

**Table 2:** T tests for difference in the averages—quantitative values of n1 (5 to 12 years of age)

	(*n*)	IMSW	IMIW	ICSW	ICIW
OB	277	67	64	54	57
NB	212	49	43	47	46
Means OB	9.56	47.04	45.3	33.76	27.53
Means NB	9.79	46.54	45.3	33.34	27.06
SD OB	1.84	3.63	4.32	4.45	2.68
SD NB	1.79	3.85	4.69	3.26	2.63
OB*	0.11	0.44	0.54	0.61	0.35
NB*	0.11	0.55	0.72	0.48	0.39
Difference of means	−0.231779	0.496497	0.002362	0.423089	0.465446
CI	95 %	95 %	95 %	95 %	95 %
IC	(−0.557135; 0.093576)	(−0.905768; 1.898762)	(−1.780811; 1.785535)	(−1.106945; 1.953123)	(−0.577590; 1.508483)
T value	−1.4	0.7	0	0.55	0.89
*p* value	0.62	0.484	0.998	0.584	0.378
DF	459	99	85	96	97

*Significant values

### Group 2 (n2 = 443) 13–18 years old—qualitative variables

For the age group 13–18 years old, there is no evidence of an association between gender, class III malocclusion, PMx and RMx, PM and RM, LM, BP and DP, and the type of respiration

There is an association between class II malocclusion, SM and ILAFH, and OB. It can be seen that there is a higher percentage of OB with these variables and that there is a higher percentage of NB with class I malocclusion and of NB with NLAFH.

**Fig. 4:**
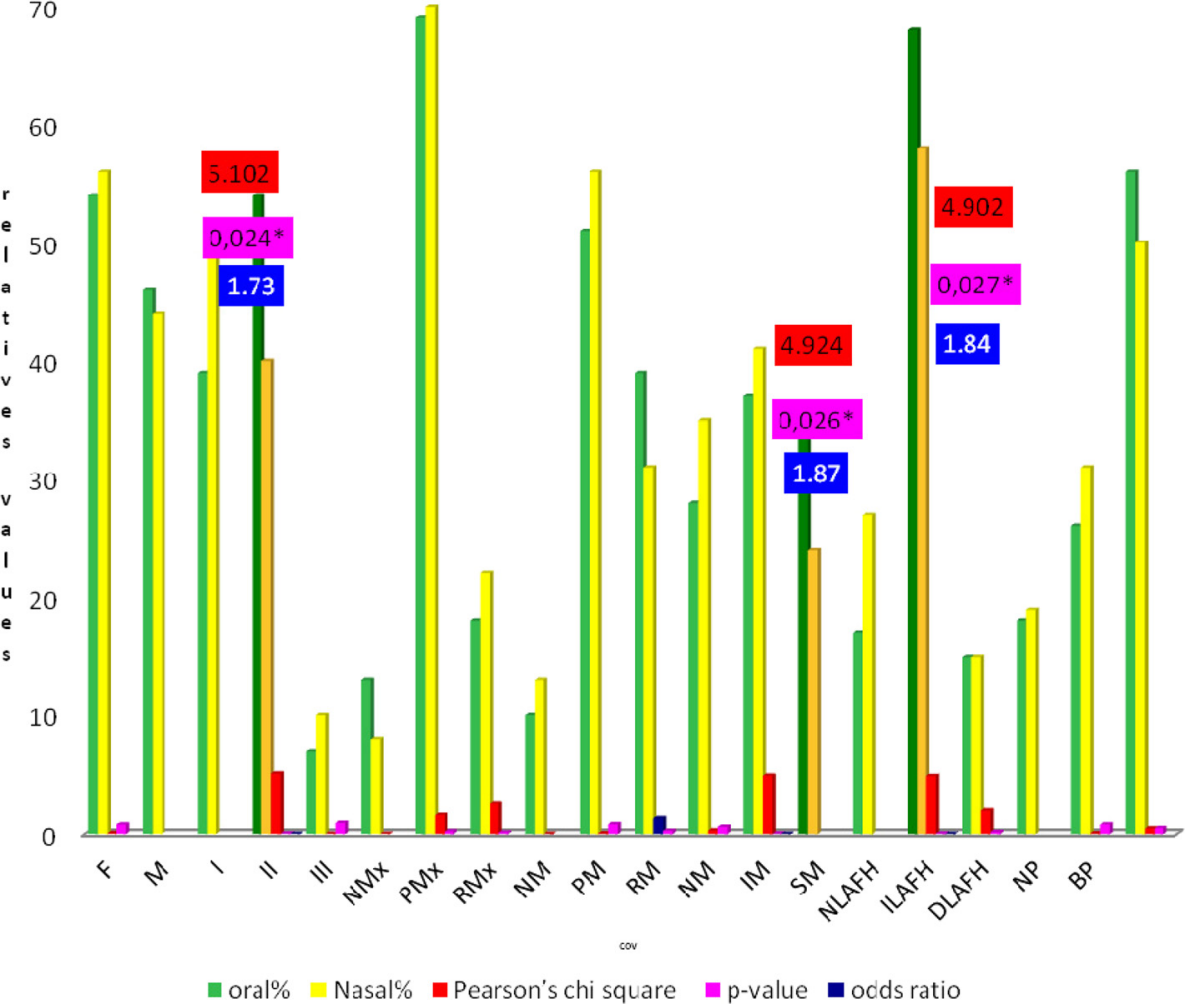
Intra-groups association values (Pearson’s *x* ^2^ odds ratio) for qualitative variables, n2 (924), 13 to 18 years of age, with oral and nasal breathing. *Significant values. The variables that showed significant associations and the risk of disease were Class II, SM and ILAFH

We calculated the OR for class II malocclusion (OR = 1.73; CI (95 %) = (1.07, 2.78), SM (OR = 1.87; CI (95 %) = (1.07, 3.24) ILAFH (OR = 1.84, CI (95 %) = (1.07, 3.17) with OB. The results of *x*^2^ and Pearson associations, OR for qualitative variables tests, in group 2 are shown in Table 3 and Fig. 4.

**Table 3:** Intra-group association tests (Pearson’s x^2^, odds ratio) of qualitative variables for the group n3 (13 to 18 years of age)

Variables	OBl(n)	OBl % NB (n)	NB %	Total	Pearson X	*p*-value DF	Lilihood x	*p*-value	DF OD	IC 95 %	DF	OR	IC 95 %
F	91	54	104	56		0.072	0.788	1	0.072	0.7888	1		
M	76	46	82	44									
**Total**	**167**		**186**		**353**								
I	52	39	85	50									
II	73	54	69	40		5.102	0.024*	1	5.121	0.024*	1	1.73	**(1.07; 2.78)**
III	10	7	17	10		0.008	0.928	1	0.008	0.928	1		
**Total**	**135**		**171**		**306**								
NMx	21	13	15	8									
PMx	113	69	127	70		1.586	0.208	1	1.59	0.207	1		
RMx	29	18	40	22		2.521	0.112	1	2.528	0.112	1		
**Total**	**163**		**182**		**345**								
NM	17	10	23	13									
PM	83	51	103	56		0.06	0.806	1	0.06	0.806	1		
RM	64	39	57	31		1.299	0.254	1	1.302	0.254	1		
**Total**	**164**		**183**		**347**								
NM	45	28	63	35									
IM	60	37	73	41		0.288	0.592	1	0.288	0.591	1		
SM	56	35	42	24		4.924	0.026*	1	4.943	0.026*	1	1.87	(1.07; 3.24)
**Total**	**161**		**178**		**339**								
NLAFH	27	17	47	27									
ILAFH	109	68	103	58		4.902	0.027*	1	4.957	0.026*	1	1.84	(1.07; 3.17)
DLAFH	25	15	26	15									
**Total**	**161**		**176**		**337**								
NP	29	18	35	19									
BP	42	26	55	31		0.063	0.801	1	0.063	0.801	1		
DP	90	56	89	50		0.465	0.495	1	0.466	0.495	1		
**Total**	**161**		**179**										

*Significant p values <0.05

### Quantitative variables

There is a significant difference between the mean age of the OB and NB for the age group 13–18 years. The OB subjects are on average 0.4 years younger than NB, with the 95 % confidence level between 0.06 and 0.8 years.

There is no evidence of a difference between the average width of the maxilla and the average width of mandible, as determined by the intermolar distances and the upper and lower intercanine distances of oral and nose breathing adolescents in the 13 to 18 age group.

Summaries of the average range values are shown in Table 4.

**Table 4:** T tests for difference in the averages—quantitative values for group n2 (13 to 18 years of age)

	(*n*)	IMSW	IMIW	ICSW	ICIW
OB	167	46	42	42	45
NB	186	49	46	45	45
Means OB	14.83	47.39	45.55	34.61	27.3
Means NB	15.25	47.02	46.1	34.5	27.81
SD OB	1.76	3.2	3.57	3.22	2.8
SD NB	1.7	3.32	4.22	3.16	2.9
OB*	0.14	0.47	0.55	0.5	0.31
NB*	0.12	0.47	0.62	0.47	0.43
Dif of means	−0.426341	0.370896	−0.550207	0.111905	−0.777778
CI	95 %	95 %	95 %	95 %	95 %
IC	(−0.789891; −0.062791)	(−0.958786; 1.700578)	(−2.203110; 1.102696)	(−1.250651; 1.474461)	(−1.837129; 0.281573)
T value	−2.31	0.55	−0.66	0.16	1.46
*p* value	0.022*	0.581	0.51	0.871	0.148
DF	343	92	85	84	79

*Significant values

### Group 3 (n3 =312) 19–57 years old—qualitative variables

For the age group 19–57 years old, there is no evidence of an association between gender, class III malocclusion, PMx and RMx, PM and RM, LM and SM, BP and DP, and mode of breathing.

For the age group of 19 to 57 years we found a marginally significant association between classification II malocclusion and oral breathing (OR = 1.72; CI (95 %) = (0.99, 2.98)).

**Fig. 5:**
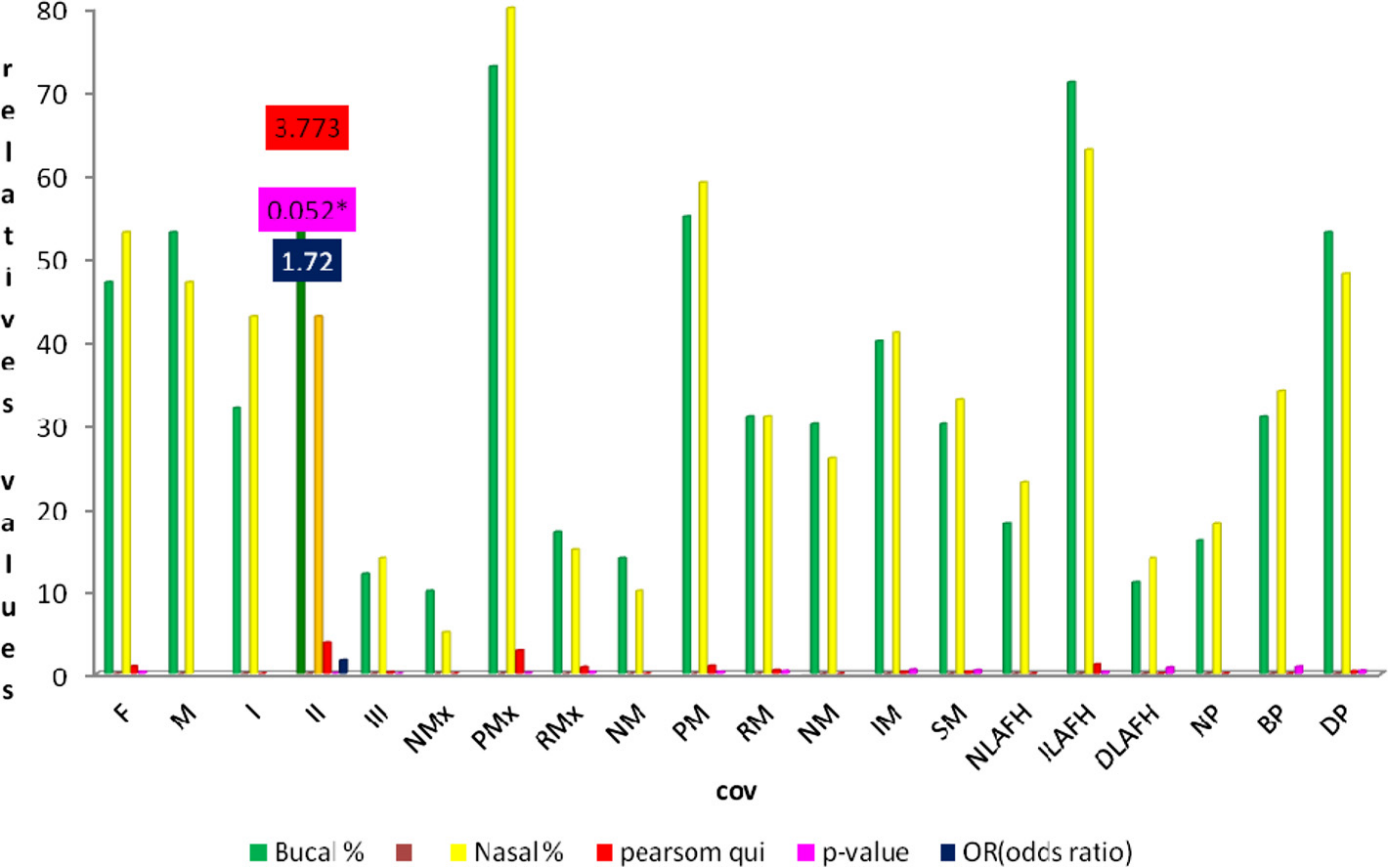
Intra-groups association values, (Pearson’s *x* ^2^, OR, for qualitative variables n3 (19 to 57 years of age), with OB and NB. *Significant values. The variable that showed significant associations and the risk of disease was the Angle Class II

The results of intra-group association tests of qualitative variables (Pearson’s chi-square and odds ratio) for the age group 19–57 years with (n3 = 312) are shown in Table 5 and Fig. 5.

**Table 5:** Intra-group association tests (Pearson’s x^2^, odds ratio) of qualitative variables—group n3 (19 to 57 years of age)

Variables	OB (*n*)	OB %	NB (*n*)	NB %	Total	Pearson *x* ^2^	*p* value	DF	Lilihood *x* ^2^	OR	CI 95 %
F	95	47	110	53		1.012	0,314	1	1.012		
M	56	53	51	47							
*Total*	*151*		*161*		*312*						
I	36	32	57	43							
II	63	56	58	43		3.773	0.052*	1	3.792	*1.72*	*(0.99; 2.98)*
III	14	12	19	14		0.14	0.708	1	0.14		
*Total*	*113*		*134*		*247*						
NMx	15	10	8	5							
PMx	107	73	122	80		2.862	0.091	1	2.894		
RMx	25	17	22	15		0.912	0.34	1	0.923		
*Total*	*147*		*152*		*299*						
NM	21	14	16	10							
PM	81	55	90	59		1.073	0.3	1	1.075		
RM	46	31	47	31		0.564	0.453	1	0.565		
*Total*	*148*		*153*		*301*						
NM	42	30	39	26							
IM	57	40	61	41		0.242	0.623	1	0.242		
SM	43	30	48	33		0.363	0.547	1	0.363		
*Total*	*142*		*148*		*290*						
NLAFH	26	18	34	23							
ILAFH	100	71	94	63		1.237	0.266	1	1.24		
DLAFH	15	11	21	14		0.026	0.873	1	0.026		
*Total*	*141*		*149*		*290*						
NP	22	16	26	18							
BP	43	31	50	34		0.002	0.964	1	0.002		
DP	74	53	70	48		0.444	0.505	1	0.445		
*Total*	*139*		*146*		285						

*Significant values

### Quantitative values

There is a significant difference between the average width of the mandibles in OB subjects in group 3. The OB have mandibles that are on average 2.4 mm wider than NB, with a 95 % confidence level (0.4 and 4.5 mm). Summaries of the values of the middle range are shown in Table 6.

**Table 6:** T test for differences of averages- Quantitative values for group n3 (19 to 57 years of age)

	(*n*)	IMSW	IMIW	ICSW	ICIW
OB	151	41	36	43	43
NB	161	43	39	37	37
Means OB	26.17	47.93	47.07	35.01	28.37
Means NB	27.95	46.7	44.65	34.86	27.64
SD OB	6.71	4.01	4.09	4.57	3.03
SD NB	7.35	4.29	4.74	3.61	2.91
OB*	0.55	0.63	0.68	0.7	0.46
NB*	0.58	0.65	0.66	0.79	0.48
Dif of means	−1.77813	1.22915	2.4156	0.146763	0.736958
CI	95 %	95 %	95 %	95 %	95 %
IC	(−3.34477; −0.21148)	(−0.57221; 3.03052)	(0.38313; 4.44807)	(−1.675761; 1.969287)	(−0.587233; 2.061149)
T value	−2.23	1.36	2.37	0.16	1.11
*p* value	0.026*	0.178	0.021*	0.873	0.271
DF	309	81	72	77	77

*Significant

There was a significant difference between the OB and NB: OB are younger, and the inferior molar widths are larger than the superior molar widths

## Discussion

We assessed skeletal and dental changes in a large group of patients, to minimize the probable random errors. Statistical tests helped to widespread the results and confirmed the associations (*x*^2^) and disease risk (OR).

OB is a result of the influence of genetic and environmental factors [4, 13]. Some authors claim that diseases such as allergies [17], chronic colds, habits [22, 27], head position [12], or shape of the upper airway [2, 7] may lead to nasal obstruction and consequent OB [1, 8, 12, 13, 16, 28]. However, the relative influence of these factors in the genesis of OB remains unclear [2, 5–7, 10, 11, 14, 20, 21]. Studies indicate that factors that cause OB do not cause malocclusion [20] or modify facial patterns [2, 5].

Most studies included children and adolescents, because more significant changes occur in these groups [1–10, 12, 16–19, 21–23, 27–29] and the changes become less apparent in adulthood [15]. Dentition and aging affect the initiation and persistence of craniofacial changes [8, 15, 18]. Alterations in early age do not persist into adulthood [17, 20]. The influence of OB on craniofacial development becomes evident from the ages of 8 to 10 years [18].

Class II malocclusion remained present in all patients with OB, as disease risk. This condition improved in adult patients in our study (n3), with only a marginal association with class II and OB [17, 20]. It seems reasonable to suggest that early treatment of that condition may be beneficial, especially in young individuals. Treatment should be multidisciplinary [14].

In our groups, we observed a higher proportion of subjects class II. Initially, we made the separation of groups by age and by mode of breathing, so the occlusal and skeletal variables were divided into groups, regardless of their initial ratio.

We obtained different results for each age group. In group n1, most children BP and with DLAFH, breathe through the nose and were class I, and these characteristics were shown to be protective factors found in the OR [18]. In group n2, we found that the oral breathers tended to be younger than nasal breathers. We did not identify sexual dimorphism in any group [18], although some studies claim a slight prevalence of OB in females [5, 13]. There were fewer changes in group n3 that exhibited only significant associations with dental changes as class II malocclusion and mandibular width suggesting an increased tendency to posterior cross bite, agreeing with many studies [2, 5, 10, 15–17, 19, 20] and disagreeing with others [1, 3]. Most patients reported “crooked teeth” as the main complain, and the information of OB and NB was obtained following the protocol [1, 2, 6, 8–11, 13, 19, 28].

Some authors postulate that facial, skeletal [1, 7, 8, 10, 13, 15, 17, 20, 28], and dental [1, 7, 8, 15, 17, 20, 22, 28] changes result from the influence of oral breathing [1, 7, 13, 29]. We confirmed this statement: In group 1, we found evidence of associations of SM and RM and OB in agreement with several studies [8, 10, 13, 15, 28], and a DLAFH and BP with NB, which appeared to be protective for disease, in disagreement with some studies [8, 10, 13, 15, 28]. In group n2 we found a clear association of SM and ILAFH agreeing with previous publications [2, 10, 16]. In group 3, we found a marginally significant association between class II malocclusion and OB, confirming the literature [8, 15, 18].

The condition of exclusive oral breathing is rare; alternation between OB and NB is much more common. The boundary between the air passages for OB and NB is small, and sometimes, even though the nasal air passages are of normal size, nasal breathing is replaced by oral [3, 16].

We could not obtain concordances in the literature for the diagnosis of OB. Few studies evaluated OB using calibrated instruments, but they are not gold standards. Even as the assessments may have been made at a time when the breathing mode was alternating [3, 12, 16, 23].

Most of the literature uses subjective information obtained through questionnaires, where the patient states his mode of breathing, and some clinical trials are not carefully constructed and do not lead to results that generate confidence [1, 2, 8–11, 13, 19, 20, 28]. Our diagnosis of OB and NB was based on clinical examination and a questionnaire [11].

## Conclusions

This study showed that dental and skeletal factors are associated with OB in children, and it seems that it becomes more severe until adolescence. But adults showed no associations between OB and skeletal factors, only in dental variables, indicating that there is no cause–effect relationship between the dental and skeletal factors and OB. The treatment of nose breathing patient should be multidisciplinary, since OB remains even when dental and skeletal factors slow down.

## Abbreviations

BP: brachycephalic facial pattern
CG: control group
CI: confidence interval
DLAFH: decreased lower anterior facial height
DP: dolichocephalic pattern
IC: intercanine
ICIW: intercanine width inf
ICSW: intercanine width sup
ILAFH: increased lower anterior facial height
IMIW: intermolar width inf
IMSW: intermolar width sup
LM: large mandible
NB: nasal breathing
NLAFH: normal lower anterior facial height
NM: normal mandible
NP: normocephalic pattern
OB: oral breathing
OR: odds ratio
OSA: sleep apnea
PM: protruded mandible
PMx: protruded maxilla
RM: retruded mandible
RMx: retruded maxilla
SD: standard deviations
SG: study group
SM: short mandible
